# Serum ergothioneine and risk of dementia in a general older Japanese population: the Hisayama Study

**DOI:** 10.1111/pcn.13893

**Published:** 2025-09-05

**Authors:** Xiangyin Meng, Tomoyuki Ohara, Kentaro Nishioka, Mao Shibata, Makoto Katsube, Norifumi Tateishi, Yumi Nakamura, Emi Oishi, Satoko Sakata, Yoshihiko Furuta, Tomohiro Nakao, Toshiharu Ninomiya

**Affiliations:** ^1^ Department of Neuropsychiatry Graduate School of Medical Sciences, Kyushu University Fukuoka Japan; ^2^ Department of Epidemiology and Public Health Graduate School of Medical Sciences, Kyushu University Fukuoka Japan; ^3^ Research Institute, Suntory Global Innovation Center Ltd. Kyoto Japan; ^4^ Department of Psychosomatic Medicine Graduate School of Medical Sciences, Kyushu University Fukuoka Japan; ^5^ Department of Center for Cohort Studies Graduate School of Medical Sciences, Kyushu University Fukuoka Japan; ^6^ Department of Medicine and Clinical Science, Graduate School of Medical Sciences, Kyushu University Fukuoka Japan

**Keywords:** Alzheimer's disease, dementia, ergothioneine, prospective studies, risk factors

## Abstract

**Aim:**

To investigate the association between serum ergothioneine and risk of developing dementia and its subtypes in a community‐dwelling older population.

**Methods:**

In this prospective longitudinal analysis of participants enrolled in the Hisayama Study, 1344 Japanese community‐residents aged 65 years and over without dementia at baseline were followed prospectively for a median of 11.2 years (2012–2023). Serum ergothioneine levels were quantified using liquid chromatography–mass spectrometry and divided into quartiles. Cox proportional hazards models were used to estimate the hazard ratios (HRs) and their 95% confidence intervals for the association between serum ergothioneine levels and the risk of dementia subtypes.

**Results:**

During the follow‐up, 273 participants developed all‐cause dementia. Among them, 201 had Alzheimer's disease (AD) and 72 had non‐Alzheimer's disease (non‐AD) dementia. The age‐ and sex‐adjusted HRs for all‐cause dementia, AD, and non‐AD dementia decreased progressively across increasing quartiles of serum ergothioneine (all *P* for trend <0.05). These associations remained significant after adjustment for a wide range of cardiovascular, lifestyle, and dietary factors, including daily vegetable intake (*P* for trend <0.05). In subgroup analyses stratified by daily vegetable intake, higher serum ergothioneine levels were consistently associated with lower dementia risk, irrespective of vegetable consumption.

**Conclusions:**

Our findings showed that higher serum ergothioneine levels were associated with a lower risk of developing all‐cause dementia, AD, and non‐AD dementia in an older Japanese population. Since ergothioneine cannot be synthesized in the human body, a diet rich in ergothioneine may be beneficial in reducing the risk of dementia.

Along with the rapid aging of the global population, the societal burden of dementia is also expanding, and addressing this problem has become a public health priority.^1^ Epidemiological investigations have already identified a dozen or so modifiable risk factors that might be used to reduce dementia risk.^2^ Non‐pharmacological therapy for dementia, specifically lifestyle modifications and dietary strategies, have attracted much attention in this regard.^3^ Dietary components, including nutrients and bioactive compounds, are known to contribute to dementia‐risk reduction through their antioxidant and anti‐inflammatory activities. Mushrooms in particular are rich in ergothioneine and other components reported to possess antioxidant and anti‐inflammatory properties,^4^ and thus might be especially effective in reducing the risk of dementia.^5^, ^6^ Indeed, recent cross‐sectional studies conducted in community‐dwelling residents reported a significant positive association between mushroom consumption and cognitive function.^7^, ^8^ Furthermore, a few population‐based prospective longitudinal studies have demonstrated significant associations between mushroom consumption and reduced risk of developing dementia, suggesting the potential benefits of mushroom components as a prophylactic against dementia.^9^, ^10^

Ergothioneine is a compound predominantly found in mushrooms and cannot be synthesized in the human body. Since the blood concentration of ergothioneine depends on its intake and is sustained for approximately one month after ingestion,^11^ it is a good candidate for a dietary approach to reducing dementia risk.^4^, ^12^ A few clinical cross‐sectional studies have reported that whole blood and plasma ergothioneine concentrations are significantly higher in cognitively normal individuals in a community than patients with dementia or mild cognitive impairment (MCI).^13^, ^14^ In addition, a prospective study with MCI individuals using data from the Alzheimer's Disease Neuroimaging Initiative (ADNI) found that those with higher blood ergothioneine levels were less likely to progress to Alzheimer's disease (AD) within 2 years than those with lower blood ergothioneine levels.^15^ However, few population‐based prospective studies have examined this association in community‐dwelling older adults, particularly with respect to the risk of developing all‐cause dementia. The purpose of the present study was to investigate the association between serum ergothioneine levels and the risk of developing dementia in an older Japanese population.

## Methods

### Study population

The Hisayama Study is a long‐term, community‐based cohort study of cerebrovascular and cardiovascular disorders that was initiated in 1961 in the town of Hisayama, located in Fukuoka Prefecture, Japan. In this study, systematic assessments targeting dementia have been conducted every 5 to 7 years since 1985.^16^ Of the 2036 individuals aged 65 years and older living in this town, a total of 1906 (comprising 1126 women and 780 men) (participation rate: 93.6%) took part in the cognitive and general health examination conducted between 2012 and 2013. After excluding 44 participants without consent to participate in this study, 339 participants with dementia at baseline, 175 participants lacking serum ergothioneine data, two participants who did not complete the baseline cognitive assessment, and two participants with intellectual disability or consciousness disturbance, the remaining 1344 participants, comprising 765 women and 579 men, were enrolled in the present study (Fig. S1). The present study was conducted in accordance with the provisions of the Declaration of Helsinki and with the approval of the Kyushu University Institutional Board of Clinical Research (approval no. 23061–04). We obtained written informed consent from all participants.

### Follow‐up surveys

The median follow‐up duration from the baseline examination was 11.2 years (interquartile range [IQR], 10.0–11.4 years). Throughout the follow‐up, neurological outcomes, such as any cognitive impairment and stroke, were collected using an established surveillance system involving the research team, community clinicians, and the town's Health Office, as reported previously.^16^ Annual health examinations were conducted to detect incident dementia cases. For participants who missed these examinations or relocated from the town, the follow‐up was supplemented with postal and telephone surveys. To enhance case ascertainment, thorough cognitive and neuropsychological evaluation for dementia was conducted in 2022–2023, involving 936 participants (69.6% of total participants who were alive at that time). Participants presenting with suspected dementia or neurological symptoms, such as cognitive impairment, underwent detailed evaluations by a psychiatrist or stroke physician involved in the study to determine a diagnosis of dementia. In cases of death, systematic investigations were conducted to identify the cause and contributing factors and detailed information was obtained through family or attending physician interviews and detailed assessment of all accessible clinical records, including neuroimaging (computed tomography/magnetic resonance imaging). Follow‐up continued until the date of neuropsychological evaluation in 2022–2023 or until November 30, 2023, for those who did not undergo the neuropsychological evaluation in 2022–2023. Apart from deceased individuals (*n* = 398), complete follow‐up data were obtained for all participants.

### Diagnosis of dementia

Dementia and mild cognitive impairment (MCI) were diagnosed based on established clinical criteria. Dementia was diagnosed according to the criteria of the Diagnostic and Statistical Manual of Mental Disorders, Third Edition, Revised,^17^ while MCI was diagnosed using the clinical criteria proposed by Petersen *et al*. in 2001.^18^ Dementia subtypes were classified as Alzheimer's disease (AD) or non‐Alzheimer's disease (non‐AD) dementia using the diagnostic criteria of the National Institute of Neurological and Communicative Disorders and Stroke and the Alzheimer's Disease and Related Disorders Association.^19^ During the neuropsychological evaluation, the Mini‐Mental State Examination (MMSE) was administered, with scores of 27 or higher considered indicative for normal cognition.^20^ When participants were suspected of having dementia or MCI, further comprehensive evaluations, including the Wechsler Memory Scale Logical Memory subtest, were conducted by expert psychiatrists.^21^ MCI was defined based on either (i) objective cognitive decline evident from the neuropsychological data or (ii) any cognitive concerns by informants (e.g., family members, the town's Health Office members, or local physicians) in the absence of clear evidence of dementia. Expert psychiatrists and stroke physicians on the study team confirmed the diagnosis of all dementia and MCI cases.

### Measurement of serum ergothioneine

In 2012–2013, we collected serum samples as part of the survey, and 95.9% of them were collected under a fasting condition. Following clot formation at room temperature (approximately 30 min), blood samples were centrifuged at 1500 g for 5 min. The isolated serum was preserved at −80°C within 3.5 to 6.0 h after collection and stored until biochemical analysis. In 2023, we thawed these serum samples. We used commercially available isotope‐labeled ergothioneine‐d9 (Toronto Research Chemicals, Toronto, Ontario, Canada) as the internal standard (IS). A 30 μL aliquot of serum was mixed with 30 μL of ultrapure water and 10 μL of a 10 μM internal standard solution, followed by the addition of 600 μL acetonitrile. This mixture was vortexed and then centrifuged at 10,000 × g for 5 min at 4°C. An aliquot of 100 μL of the resulting supernatant was collected and diluted with an equal volume (100 μL) of mobile phase A (details below). A 2 μL portion of the diluted solution was subjected to ultra‐high‐performance liquid chromatography (UHPLC) using the Shimazu LC‐20AD platform, coupled with a QTRAP5500 tandem quadrupole mass spectrometer (AB Sciex, Tokyo, Japan) for analysis.

Ergothioneine was separated chromatographically on a ZIC‐cHILIC column (3 μm, 150 × 2.1 mm, 100 Å; Merck Millipore Corporation, Burlington, MA, USA) with aqueous 0.1% formic acid as mobile phase A and acetonitrile containing 0.1% formic acid as mobile phase B. Gradient elution was performed at a flow rate of 0.4 mL/min as follows: from 0 to 0.5 min, 5% A/95% B; from 0.5 to 10 min, 5% A/95% B to 80% A/20% B; from 10 to 11 min, 80% A/20% B; from 11 to 11.1 min, 80% A/20% B to 5% A/95% B; from 11.1 to 15 min, 5% A/95% B. The mass spectrometer was operated in multiple reaction monitoring modes with positive electrospray ionization. The monitored mass transitions were m/z 230.0 to 86.4 for ergothioneine and m/z 239.0 to 195.0 for ergothioneine‐d9. Instrument parameters were set as follows: curtain gas, 30 psi; collision gas, 5 psi; source temperature, 400 °C; Gas1 and Gas2, 30 psi each; and declustering potential, 50 V.

Serum ergothioneine concentrations were quantified using weighted least square linear regression (weighting factor: 1/x^2^) based on the peak area ratio of ergothioneine to the internal standard, as derived from a calibration curve. The calibration curve included concentrations of 0.1, 0.2, 0.5, 1, 2, 5, and 10 μM. Samples exceeding the upper limit of the calibration range were appropriately diluted prior to reanalysis. Samples with concentrations below the minimum standard concentration were treated as 0.09 μM for subsequent analysis. Serum ergothioneine levels were categorized into quartile categories: <0.410, 0.410–0.692, 0.693–1.229, and >1.229 μmol/L.

### Risk factor measurements

At the baseline survey, participants completed a self‐administered questionnaire covering lifestyle factors and medical history which included educational status, smoking and drinking habits, comorbid conditions, and treatment for diabetes mellitus, hypertension, and hypercholesterolemia. Trained interviewers assisted in collecting these data. Low education was defined as having 9 or fewer years of formal education. We measured blood pressure three times in the seated position following a rest period of at least 5 min, and the mean value of the three measurements was used for analysis. Hypertension was defined as either systolic/diastolic blood pressure ≥ 140/90 mmHg or current use of antihypertensive medication. Plasma glucose levels were measured by using the hexokinase method. Diabetes mellitus was defined by one or more of the following: fasting glucose level ≥7.0 mmol/L, casual or 2‐h postload glucose level after 75‐g oral glucose tolerance test ≥ 11.1 mmol/L, or use of glucose‐lowering agents. Serum total cholesterol was measured enzymatically. Hypercholesterolemia was defined as serum cholesterol ≥5.69 mmol/L and/or use of lipid‐lowering agents. History of stroke and history of cerebrocardiovascular disease were determined based on all clinical data from the Hisayama Study. Participants' height and weight were measured under standardized conditions (light clothing, no shoes), and body mass index (BMI; kg/m^2^) was computed accordingly. Electrocardiogram abnormalities were defined according to the Minnesota Code (specifically codes 3–1, 4–1, 4–2, 4–3, and 8–3).^22^ Smoking and alcohol consumption were each categorized as current habitual use or not. We defined regular exercise as engaging in sports or other physical exercise including recreational walking at least three times per week during leisure time. A dietary survey was conducted using a Semi‐Quantitative Food Frequency Questionnaire concerning food intake.^23^ Nutritional intake was calculated using the Standard Tables of Food Composition in Japan 2015.^24^ To determine the APOE‐ε4 carrier, we genotyped two single nucleotide polymorphisms (rs429358 and rs7412) using the multiplex polymerase chain reaction‐based Invader assay^25^ or the multiplex polymerase chain reaction‐based targeted sequencing method^26^ as previously reported.

### Statistical analysis

Serum ergothioneine were log‐transformed to reduce skewness in the distribution for the analysis. Logistic regression for categorical variables and linear regression for continuous variables were used to estimate the age‐ and sex‐adjusted frequencies or mean values of risk factors across the quartiles, respectively. We tested trends in the baseline characteristics across quartiles of serum ergothioneine levels by using logistic or linear regression analysis. The incidence rate of all‐cause dementia and its subtypes were calculated using the person‐year method. Adjusted cumulative incidence of all‐cause dementia across serum ergothioneine levels was estimated using a Cox proportional hazards model that included age and sex. Separate Cox proportional hazards regression models were employed to calculate the hazard ratios (HRs) and their 95% confidence intervals (CIs) for the association between serum ergothioneine levels and the risk of dementia. In this analysis, three different models were evaluated: (1) model 1, adjusted for age and sex; (2) model 2, adjusted for age and sex (the covariates in model 1) plus low education, systolic blood pressure, antihypertensive medication, diabetes mellitus, serum total cholesterol, BMI, electrocardiogram abnormalities, history of stroke, smoking habits, alcohol intake, and regular exercise; and (3) model 3, adjusted for the covariates included in model 2 plus daily vegetable intake. We verified the proportional hazards assumption by visually inspecting the log cumulative hazard plots. Linear trends in dementia risk across ergothioneine quartiles were assessed by entering quartile values as a continuous variable in the model. Restricted cubic splines were used to show the shape of these associations with four knots placed at the 5th, 35th, 65th and 95th percentiles of log‐transformed serum ergothioneine levels (−1.58, −0.63, −0.01, and 1.08 of log‐transformed serum ergothioneine, respectively). The fifth percentile was set as the reference value. Non‐linearity was assessed by comparing model fit between linear and spline models using the likelihood ratio test. We analyzed the risk estimates per 1‐standard deviation (SD) increase in log‐transformed serum ergothioneine levels by using a model that included log‐transformed serum ergothioneine levels as a continuous variable. To assess the heterogeneity of the association across subgroups, multiplicative interaction terms were added to the relevant model.^27^ We performed sensitivity analyses by censoring participants who developed dementia within the first 2 years of follow‐up (*n* = 39). In addition, we analyzed competing risks of death using the Fine–Gray subdistribution hazards model.^28^ To examine whether the association between serum ergothioneine and risk of dementia is independent of the influence of vegetable intake, we conducted a subgroup analysis stratified by daily vegetable intake, in which serum ergothioneine levels and the levels of daily vegetable intake were divided into two groups based on the median value for each (0.693 μmol/L and 165.0 g/day, respectively) and the four groups were classified by combining these groups. In addition, we conducted a subgroup analysis of the association between serum ergothioneine levels and the risk of dementia by stratifying participants based on the presence or absence of MCI at baseline. The software package SAS version 9.4 (SAS Institute Inc., Cary, NC, USA) was used to perform all statistical analyses, and statistical significance was set at a two‐tailed *P*‐value of <0.05 in all analyses.

## Results

The median age was 73 years (IQR: 69–79), and the oldest participant was 100 years old. The median of serum ergothioneine was 0.693 μmol/L (IQR 0.409–1.229). Table 1 shows age‐ and sex‐adjusted baseline characteristics according to the total and quartiles of serum ergothioneine concentration. The frequencies of female gender, habitual exercise, and drinking habits, as well as the mean values of BMI and daily vegetable intakes for green and yellow vegetables and for other vegetables, increased significantly with higher serum ergothioneine levels. Meanwhile, the frequencies of smoking habits and history of stroke and the mean values of age decreased significantly with higher serum ergothioneine levels.

**Table 1:** Age‐ and sex‐adjusted baseline characteristics of participants according to the quartile of serum ergothioneine, 2012–2013

Variable	Total population (*n* = 1344)	Serum ergothioneine (μmol/L)				*P* for trend
		Q1 (<0.410)	Q2 (0.410–0.692)	Q3 (0.693–1.229)	Q4 (>1.229)	
		(*n* = 335)	(*n* = 337)	(*n* = 335)	(*n* = 337)	
Age, years	74.2 (0.2)	75.6 (0.4)	74.3 (0.4)	73.5 (0.4)	73.3 (0.4)	<0.001
Female, %	56.9	54.3	50.1	59.0	64.3	0.002
Education ≤9 years, %	37.3	42.9	35.0	37.3	34.2	0.05
Systolic blood pressure, mmHg	134.8 (0.5)	135.2 (1.0)	134.3 (1.0)	135.5 (1.0)	134.1 (1.0)	0.64
Diastolic blood pressure, mmHg	76.5 (0.3)	76.9 (0.6)	76.0 (0.6)	77.0 (0.6)	76.1 (0.6)	0.59
Antihypertensive medication, %	56.2	52.7	55.1	57.1	59.7	0.07
Hypertension, %	72.4	71.6	71.8	73.7	72.6	0.67
Diabetes mellitus, %	23.7	20.8	25.7	21.8	26.2	0.24
Serum total cholesterol, mg/dL	196.9 (0.9)	196.6 (1.8)	195.8 (1.8)	197.9 (1.8)	197.4 (1.8)	0.60
Body mass index, kg/m^2^	23.1 (0.1)	22.6 (0.2)	23.3 (0.2)	23.3 (0.2)	23.4 (0.2)	0.002
Electrocardiogram abnormalities, %	16.2	18.5	17.3	14.9	14.0	0.08
History of stroke, %	5.3	7.8	5.4	3.5	4.5	0.04
Smoking habits, %	5.8	10.4	7.2	3.5	2.5	<0.001
Alcohol intake, %	38.2	30.6	38.1	44.6	39.6	0.01
Regular exercise, %	39.6	35.6	36.0	42.4	44.3	0.01
Daily total energy intake, kcal/day	1536.9 (8.9)	1528.5 (18.2)	1552.6 (17.8)	1533.8 (17.6)	1532.4 (17.6)	0.92
Daily vegetable intake, g/day	170.0 (2.1)	158.7 (4.2)	162.8 (4.1)	178.8 (4.0)	178.7 (4.0)	<0.001
Green and yellow vegetables	56.9 (0.8)	52.9 (1.6)	54.7 (1.5)	59.7 (1.5)	60.0 (1.5)	<0.001
Other vegetables	112.8 (1.4)	105.4 (2.9)	107.5 (2.8)	119.0 (2.8)	118.6 (2.8)	<0.001
Mild cognitive impairment, %	11.9	13.9	12.1	11.6	10.0	0.12
APOE‐ε4 allele career, %	17.9	17.7	16.2	19.3	18.4	0.58

*Note*: Electrocardiogram abnormalities were defined as Minnesota Code 3–1, 4–1, 4–2, 4–3, or 8–3. Data are presented as mean values (standard error) or proportion.

Abbreviations: APOE, Apolipoprotein E; MMSE, Mini‐Mental State Examination.

During a median follow‐up of 11.2 (IQR 10.0–11.4) years, 273 participants (172 women and 101 men) developed all‐cause dementia. Of these, 196 underwent brain imaging, 32 underwent autopsy, and 31 underwent both procedures; hence, 197 (72.2%) underwent some kind of morphological examination. Regarding the subtypes of dementia, 201 participants developed AD, and 72 developed non‐AD dementia. The age‐ and sex‐adjusted cumulative incidence of all‐cause dementia decreased significantly with elevating serum ergothioneine levels (*P* for trend <0.001; Fig. S2).

Table 2 shows crude incidence rates, and the age‐ and sex‐adjusted and multivariable‐adjusted HRs and 95% CIs for the risk of all‐cause dementia and its subtypes by serum ergothioneine levels. Crude incidence rates of all‐cause dementia and its subtypes declined linearly with higher serum ergothioneine levels. The age‐ and sex‐adjusted HRs of all‐cause dementia and its subtypes decreased significantly with higher serum ergothioneine levels (all *P* for trend <0.05) (model 1). These associations were unchanged after adjusting for age, sex, education status, systolic blood pressure, antihypertensive medication, diabetes mellitus, serum total cholesterol, BMI, electrocardiogram abnormalities, history of stroke, smoking habits, alcohol intake, and regular exercise (model 2). Moreover, when adjusting for daily vegetable intake in addition to the above‐mentioned covariates (model 3), the observed significant associations did not change substantially. Sensitivity analyses censoring incident dementia cases within 2 years of follow‐up demonstrated a similar significant association between serum ergothioneine levels and risk of all‐cause dementia (Table S1). When assessing the association between serum ergothioneine levels and risk of mortality, no significant association with the risk of mortality was observed (Table S2). In addition, when we conducted a competing risk analysis by using the Fine–Gray subdistribution hazards model, which treated death as a competing risk, the observed results did not change substantially (Table S1).

**Table 2:** Association between serum ergothioneine levels and risk of dementia and its subtypes, 2012–2023

Serum ergothioneine levels (μmol/L)	No. of events/PYs	Crude incidence rate (per 10^3^ PYs)	Hazard ratio (95% confidence interval)					
			Model 1 (Age‐ and sex‐adjusted)	*P* for trend	Model 2 (Multivariable‐adjusted^†^)	*P* for trend	Model 3 (Multivariable‐adjusted^‡^)	*P* for trend
All‐cause dementia								
Q1 (<0.410)	92/3007	30.6	1.00 (reference)		1.00 (reference)		1.00 (reference)	
Q2 (0.410–0.692)	78/3217	24.2	0.89 (0.66–1.21)		0.94 (0.69–1.28)		0.97 (0.69–1.35)	
Q3 (0.693–1.229)	57/3354	17.0	0.69 (0.50–0.96)		0.70 (0.50–0.98)		0.72 (0.51–1.03)	
Q4 (>1.229)	46/3443	13.4	0.54 (0.38–0.78)	<0.001	0.55 (0.38–0.80)	<0.001	0.56 (0.38–0.84)	0.002
Alzheimer's disease								
Q1 (<0.410)	68/3007	22.6	1.00 (reference)		1.00 (reference)		1.00 (reference)	
Q2 (0.410–0.692)	53/3217	16.5	0.84 (0.58–1.20)		0.88 (0.61–1.27)		0.82 (0.55–1.23)	
Q3 (0.693–1.229)	42/3354	12.5	0.70 (0.48–1.03)		0.69 (0.47–1.02)		0.70 (0.47–1.06)	
Q4 (>1.229)	39/3443	11.3	0.63 (0.43–0.94)	0.01	0.62 (0.41–0.94)	0.01	0.61 (0.39–0.94)	0.02
Non‐Alzheimer's disease dementia								
Q1 (<0.410)	24/3007	8.0	1.00 (reference)		1.00 (reference)		1.00 (reference)	
Q2 (0.410–0.692)	25/3217	7.8	1.04 (0.59–1.83)		1.10 (0.62–1.95)		1.43 (0.77–2.66)	
Q3 (0.693–1.229)	15/3354	4.5	0.67 (0.35–1.27)		0.72 (0.37–1.42)		0.78 (0.38–1.62)	
Q4 (>1.229)	7/3443	2.0	0.31 (0.13–0.71)	0.003	0.35 (0.15–0.82)	0.01	0.43 (0.17–1.04)	0.03

Abbreviation: PYs, person‐years.

^†^ Model 2: Adjusted for age, sex, education status, systolic blood pressure, antihypertensive medication, diabetes mellitus, serum total cholesterol, body mass index, electrocardiogram abnormalities, history of stroke, smoking habits, alcohol intake, and regular exercise.

^‡^ Model 3: Adjusted for the covariates included in model 2 plus daily vegetable intake.

**Fig. 1:**
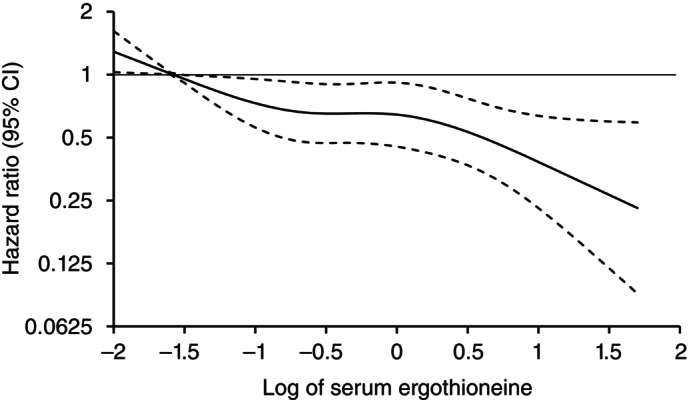
Restricted cubic splines for the association between serum ergothioneine levels and risk of all‐cause dementia. Solid lines represent the hazard ratios; dashed lines represent the 95% confidence intervals. Knots were placed at the 5th, 35th, 65th and 95th percentiles (−1.58, −0.63, −0.01 and 1.08) of log‐transformed serum ergothioneine. A reference point was set at the 5th percentile of log‐transformed serum ergothioneine. Serum ergothioneine values over the 99th percentile were not included in the plots. The *P*‐value for non‐linearity was 0.35 for all‐cause dementia. The risk estimates were adjusted for age, sex, education status, systolic blood pressure, antihypertensive medication, diabetes mellitus, serum total cholesterol, body mass index, electrocardiogram abnormalities, history of stroke, smoking habits, alcohol intake, regular exercise and daily vegetable intake.

**Fig. 2:**
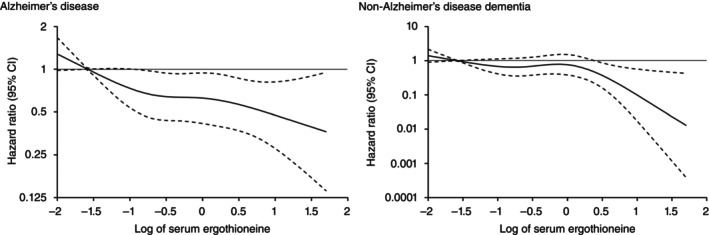
Restricted cubic splines for the association between serum ergothioneine levels and risk of dementia subtypes. Solid lines represent the hazard ratios; dashed lines represent the 95% confidence intervals. Knots were placed at the 5th, 35th, 65th and 95th percentiles (−1.58, −0.63, −0.01 and 1.08) of log‐transformed serum ergothioneine. A reference point was set at the 5th percentile of log‐transformed serum ergothioneine. Serum ergothioneine values over the 99th percentile were not included in the plots. The *P*‐values for non‐linearity were 0.22 for Alzheimer's disease and 0.59 for non‐Alzheimer's disease dementia. The risk estimates were adjusted for age, sex, education status, systolic blood pressure, antihypertensive medication, diabetes mellitus, serum total cholesterol, body mass index, electrocardiogram abnormalities, history of stroke, smoking habits, alcohol intake, regular exercise and daily vegetable intake.

Figure 1 shows the associations between serum ergothioneine levels and the risk of all‐cause dementia, as analyzed using a restricted cubic spline analysis. The risks of developing all‐cause dementia decreased approximately linearly as serum ergothioneine levels increased (*P* for non‐linearity = 0.35). Decreasing risks of developing both AD and non‐AD dementia with higher serum ergothioneine levels were also observed (Fig. 2).

We estimated the multivariable‐adjusted HRs of developing all‐cause dementia per log‐transformed 1‐SD increment in the serum ergothioneine levels in subgroups with other potential risk factors for dementia (Table 3). With regard to the subgroups of age and sex, the association between serum ergothioneine levels and the risk of dementia tended to be weaker in the older age group and in women (both *P* for heterogeneity <0.10). However, the risk of dementia generally decreased with higher serum ergothioneine levels, regardless of age and sex subgroup (all *P*‐values <0.10). On the other hand, the decrease in dementia risk associated with higher serum ergothioneine levels was consistently observed across all subgroups of other risk factors except for obesity, smoking habits, and APOE‐ε4 carriage, without evidence of heterogeneity (all *P* for heterogeneity >0.10). With regard to subgroups of obesity, smoking, and APOE‐ε4 carriage, no significant associations were observed between serum ergothioneine levels and dementia risk; however, there was also no evidence of significant heterogeneities across these subgroups.

**Table 3:** Hazard ratios of dementia per 1‐SD increment in serum ergothioneine level in various subgroups, 2012–2023

Variables	Events, *n*	Person‐years	Crude incidence rate/1000 person‐years	Hazard ratio (95% CI), p value	*P* for heterogeneity
Overall	238	12,299	19.4	0.75 (0.65–0.86), *P* < 0.001	
Age					
≺75 years	94	8673	10.8	0.66 (0.53–0.82), *P* < 0.001	0.07
≥75 years	144	3626	39.7	0.83 (0.69–0.998), *P* = 0.048	
Sex					
Men	94	5118	18.4	0.58 (0.45–0.76), *P* < 0.001	0.047
Women	144	7181	20.1	0.84 (0.71–0.998), *P* = 0.048	
Education level					
≤9 years	113	4193	26.9	0.82 (0.67–1.01), *P* = 0.06	0.37
>10 years	125	8106	15.4	0.72 (0.59–0.87), *P* = 0.001	
Hypertension					
No	51	3919	13.0	0.68 (0.50–0.92), *P* = 0.01	0.44
Yes	187	8380	22.3	0.77 (0.65–0.90), *P* = 0.001	
Diabetes mellitus					
No	169	9444	17.9	0.80 (0.68–0.95), *P* = 0.01	0.15
Yes	69	2855	24.2	0.63 (0.48–0.83), *P* < 0.001	
Obesity					
No	179	9004	19.9	0.72 (0.61–0.85), *P* < 0.001	0.39
Yes	59	3295	17.9	0.85 (0.63–1.14), *P* = 0.27	
Hypercholesterolemia					
No	106	5229	20.3	0.79 (0.63–0.98), *P* = 0.03	0.35
Yes	132	7070	18.7	0.71 (0.59–0.86), *P* < 0.001	
Electrocardiogram abnormalities					
No	193	10,365	18.6	0.77 (0.66–0.90), *P* = 0.001	0.97
Yes	45	1934	23.3	0.68 (0.49–0.96), *P* = 0.03	
History of stroke					
No	229	11,738	19.5	0.76 (0.66–0.88), *P* < 0.001	0.18
Yes	9	561	16.0	0.12 (0.02–0.71), *P* = 0.02	
Smoking habits					
No	226	11,334	19.9	0.74 (0.64–0.86), *P* < 0.001	0.24
Yes	12	965	12.4	1.33 (0.63–2.77), *P* = 0.45	
Alcohol intake					
No	150	7084	21.2	0.74 (0.62–0.88), *P* < 0.001	0.75
Yes	88	5215	16.9	0.76 (0.59–0.97), *P* = 0.03	
Regular exercise					
No	146	7120	20.5	0.80 (0.67–0.96), *P* = 0.02	0.17
Yes	92	5179	17.8	0.66 (0.52–0.85), *P* < 0.001	
Daily vegetable intake level					
<165.0 g/day	115	5952	19.3	0.71 (0.57–0.87), *P* < 0.001	0.47
≥165.0 g/day	123	6347	19.4	0.80 (0.66–0.97), *P* = 0.02	
APOE‐ε4 status					
Noncarrier	175	9730	18.0	0.72 (0.61–0.85), *P* < 0.001	0.25
Carrier	56	2056	27.2	0.93 (0.69–1.26), *P* = 0.63	

*Note*: Hazard ratio and its 95% CI represent the risk of all‐cause dementia per 1‐SD increment in log‐transformed serum ergothioneine levels, where the SD of log‐transformed serum ergothioneine levels was 0.83. The risk estimates were adjusted for age, sex, education status, hypertension, diabetes, obesity, hypercholesterolemia, electrocardiogram abnormalities, history of stroke, smoking habits, alcohol intake, regular exercise, and daily vegetable intake, where the variables relevant to the subgroup were excluded from the corresponding model.

Abbreviations: CI, confidence interval; SD, standard deviation.

**Fig. 3:**
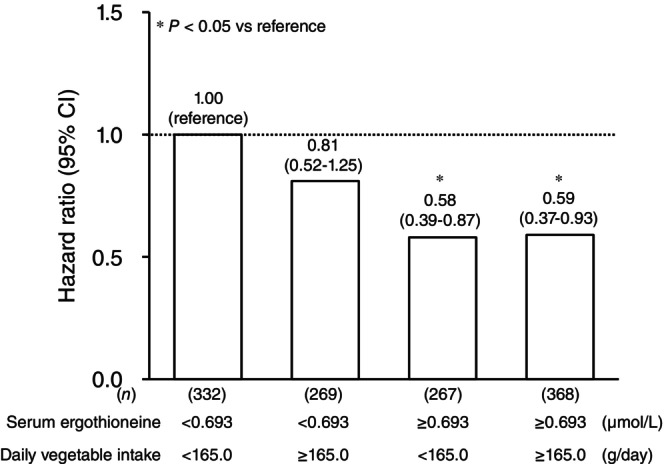
Hazard ratios of developing dementia according to serum ergothioneine levels and total vegetable intake. The risk estimates were adjusted for age, sex, education status, systolic blood pressure, antihypertensive medication, diabetes mellitus, serum total cholesterol, body mass index, electrocardiogram abnormalities, history of stroke, smoking habits, alcohol intake, regular exercise, and total daily vegetable intake.

Since vegetable intake may influence the association between serum ergothioneine levels and risk of dementia, we further examined the combined influence of serum ergothioneine levels and vegetable intake on the risk of developing all‐cause dementia (Fig. 3). Compared to the participants with low vegetable intake and a low serum ergothioneine level, the multivariable‐adjusted risks of dementia decreased significantly in those with a high serum ergothioneine level, irrespective of daily vegetable intake levels. In the subgroup analysis of daily vegetable intake levels, higher serum ergothioneine levels taken as a continuous variable were significantly associated with lower risk of dementia both in the subgroup with daily vegetable intake level of <165.0 and that with intake of ≥165.0 g/day (Table 3).

Finally, in the subgroup analysis stratified by MCI status at baseline (Table S3), higher serum ergothioneine levels were similarly associated with a lower risk of progression to dementia among participants with MCI.

## Discussion

This prospective longitudinal study demonstrated that higher serum ergothioneine levels were linearly associated with a reduced risk of developing all‐cause dementia, AD, and non‐AD dementia in a general older Japanese population without dementia. These associations did not change substantially when censoring participants with incident dementia within 2 years of follow‐up. In addition, higher serum ergothioneine levels were similarly associated with a lower risk of dementia among participants with MCI at baseline. These findings highlighted that participants with high serum ergothioneine levels were at lower risk of developing dementia and its subtypes than those with lower serum ergothioneine levels.

Ergothioneine is a dietary antioxidant abundantly found in many edible mushrooms, including oyster mushrooms, maitake mushrooms, and porcini mushrooms.^4^, ^29^ Previous prospective longitudinal studies have reported that higher mushroom intake is associated with a reduced risk of dementia.^9^, ^10^ Regarding blood ergothioneine levels, clinical studies conducted in hospital outpatients and community‐dwelling residents have shown that whole blood and plasma ergothioneine levels were significantly higher in participants with normal cognition than those with dementia.^13^, ^14^ A longitudinal study on individuals with MCI concluded that higher blood ergothioneine levels are significantly associated with a reduced risk of progression to AD within 2 years.^15^ In addition, a previous randomized controlled trial in individuals without dementia demonstrated that participants with ergothioneine supplementation exhibited a greater improvement in processing speed than those without ergothioneine supplementation after 12 weeks of follow‐up.^30^ Finally, two prospective longitudinal studies reported that higher vegetable intake is associated with a reduced risk of dementia.^31^, ^32^ There was thus a chance that the significant negative associations between serum ergothioneine levels and dementia risk observed in the present study were merely a reflection of vegetable intake levels. However, even in our multivariable‐adjusted analysis that included daily vegetable intake and in our subgroup analysis of daily vegetable intake levels, a significant association was observed between higher serum ergothioneine levels and reduced risk of dementia. Collectively, the above findings support the results of this study, suggesting that mushroom and ergothioneine intake may be useful for reducing dementia risk.

In this study, higher levels of serum ergothioneine were significantly associated with a lower risk of not only AD, but also non‐AD dementia. Ergothioneine has been reported to be both an antioxidant and anti‐inflammatory factor,^5^, ^6^ both of which are known to confer protection against developing dementia. Experimental studies using mice have reported that ergothioneine supplementation reduced amyloid β accumulation by attenuating amyloid β‐induced apoptosis.^33^, ^34^ In addition, other experimental studies suggested that ergothioneine might play a role in the protection of endothelial cells.^35^, ^36^ If so, it seems reasonable that ergothioneine might also slow the progression of cerebral small or large vessel diseases. In consideration of all the above, it is biologically plausible that ergothioneine may contribute to reducing the risk of developing dementia by mitigating the risk of neurodegeneration and cerebral vessel diseases. Further clinical and fundamental research studies are warranted to accumulate additional evidence on the association between ergothioneine and the risk of dementia.

In the subgroup analysis, the association between serum ergothioneine levels and the risk of dementia tended to be weaker in older participants and in women. In older individuals, the cumulative burden of multiple risk factors—such as hypertension, diabetes mellitus, and smoking—may contribute to both neurodegenerative and vascular pathology, potentially diminishing the relative influence of ergothioneine.^37^ In women, postmenopausal hormonal changes, particularly the decline in estrogen, have been associated with increased oxidative stress and a higher vulnerability to neurodegenerative changes.^38^, ^39^ These findings suggest that the potential benefit of ergothioneine may be attenuated in individuals with pre‐existing, multifactorial risk profiles for dementia. In addition, there was no evidence of significant negative associations between serum ergothioneine levels and dementia risk in the obesity, smoking or APOE‐ε4 carriage subgroups, although heterogeneities were not detected. The exact reason for these findings was unclear, but it may be simply that the number of participants in these groups was small, or that the favorable effect of ergothioneine on dementia was muted by the high‐level dementia risk in these groups. Further large‐scale prospective studies are warranted to examine the association between serum ergothioneine levels and the risk of dementia in more detail.

The strengths of this study include its population‐based longitudinal design, its high participation and perfect follow‐up rates, and its use of brain imaging and morphological data from autopsies to diagnose dementia subtypes. However, several limitations should be noted. First, since serum ergothioneine levels and other risk factors were measured only at baseline, we could not evaluate the changes of serum ergothioneine levels during the follow‐up period. Lifestyle modifications during follow‐up could have influenced serum ergothioneine levels and other risk factors. In addition, the serum ergothioneine level was measured only once, and from a sample. Because the samples used in this study were stored at −80°C for approximately 10 years, it is possible that degradation occurred during the storage period, potentially lowering serum concentrations. However, any such degradation would likely have affected all samples similarly, and the measured serum ergothioneine concentrations were generally consistent with those reported in a previous study conducted in a Japanese population.^40^ Nonetheless, if present, degradation could have led to misclassification of serum ergothioneine levels or other risk factors, which would likely weaken the association between serum ergothioneine levels and risk of dementia. Second, we cannot rule out residual confounding factors, such as other nutrients in mushrooms and socioeconomic status. Third, there is a possibility that dementia cases at the prodromal stage were included among the participants with low serum ergothioneine levels at baseline. However, sensitivity analyses censoring dementia cases occurring within the first 2 years of follow‐up and excluding participants with MCI at baseline did not materially alter any of the results. Fourth, we are unable to specify which mushroom varieties were predominantly consumed by participants in the town of Hisayama. Fifth, given the limited discriminative ability of serum ergothioneine and the potential degradation due to long‐term sample storage, we were unable to explore a clinically meaningful threshold value of serum ergothioneine. Sixth, the generalizability of the findings was limited because participants of this study were recruited from one town in Japan.

In conclusion, this study demonstrated that higher serum ergothioneine levels were significantly associated with a reduced risk of developing all‐cause dementia, AD, and non‐AD dementia in a general older Japanese population. Since ergothioneine cannot be synthesized in the human body, a diet rich in ergothioneine may be beneficial in reducing dementia risk. Further evidence from large‐scale population‐based prospective studies or interventional studies will be required to substantiate the findings of this study.

## Disclosure statement

Tomohiro Nakao and Tomoyuki Ohara are members of the Editorial Board of *Psychiatry and Clinical Neurosciences* and co‐authors of this article. To minimize bias, they were excluded from all editorial decision‐making related to the acceptance of this article for publication. Toshiharu Ninomiya received research grants from Suntory Holdings, Ltd., Japan. Kentaro Nishioka, Makoto Katsube, Norifumi Tateishi, and Yumi Nakamura are employees of Suntory Global Innovation Center, Ltd., and they contributed to the measurement of serum ergothioneine levels. The other authors declare that they have no conflicts of interest to disclose.

## Author contributions

XM and T. Ninomiya contributed to the study conception and study design; XM, TO, and T. Ninomiya contributed to the data analysis; XM, TO, EO, SS, YF, MS, and T. Ninomiya contributed to the data collection; KN, MK, NT, and YN contributed to the data measurement; XM, TO, KN, MS, MK, NT, YN, EO, SS, YF, T. Nakao, and T. Ninomiya contributed to the data interpretation. XM wrote the first draft of the manuscript and all authors contributed to critical revision of the manuscript.

## Supporting information


**Figure S1.** Flow chart of participants excluded at baseline in the Hisayama Study, 2012–2013.

**Figure S2.** Age‐ and sex‐adjusted cumulative incidence of all‐cause dementia according to quartiles of serum ergothioneine levels.

**Table S1.** Sensitivity analyses of the association between serum ergothioneine levels and the risk of all‐cause dementia.

**Table S2.** Association between serum ergothioneine levels and risk of all‐cause death.

**Table S3.** Association between serum ergothioneine levels and risk of all‐cause dementia stratified by MCI status at baseline, 2012–2023.

## Data Availability

The datasets used in the present study are not publicly available, because they contain confidential clinical data on the study participants. However, the data are available on reasonable request and with the permission of the Principal Investigator of this study, Toshiharu Ninomiya.
