# Supplementary material for: Serum ergothioneine and risk of dementia in a general older Japanese population: the Hisayama Study
Source: Psychiatry Clin Neurosci. 2025 Sep 5;79(12):808–16. doi: 10.1111/pcn.13893 (PMC12683611; doi:10.1111/pcn.13893)
Supplement: Supplementary file 1 — Figure S1. Flow chart of participants excluded at baseline in the Hisayama Study, 2012–2013. [file PCN-79-808-s005.pdf]

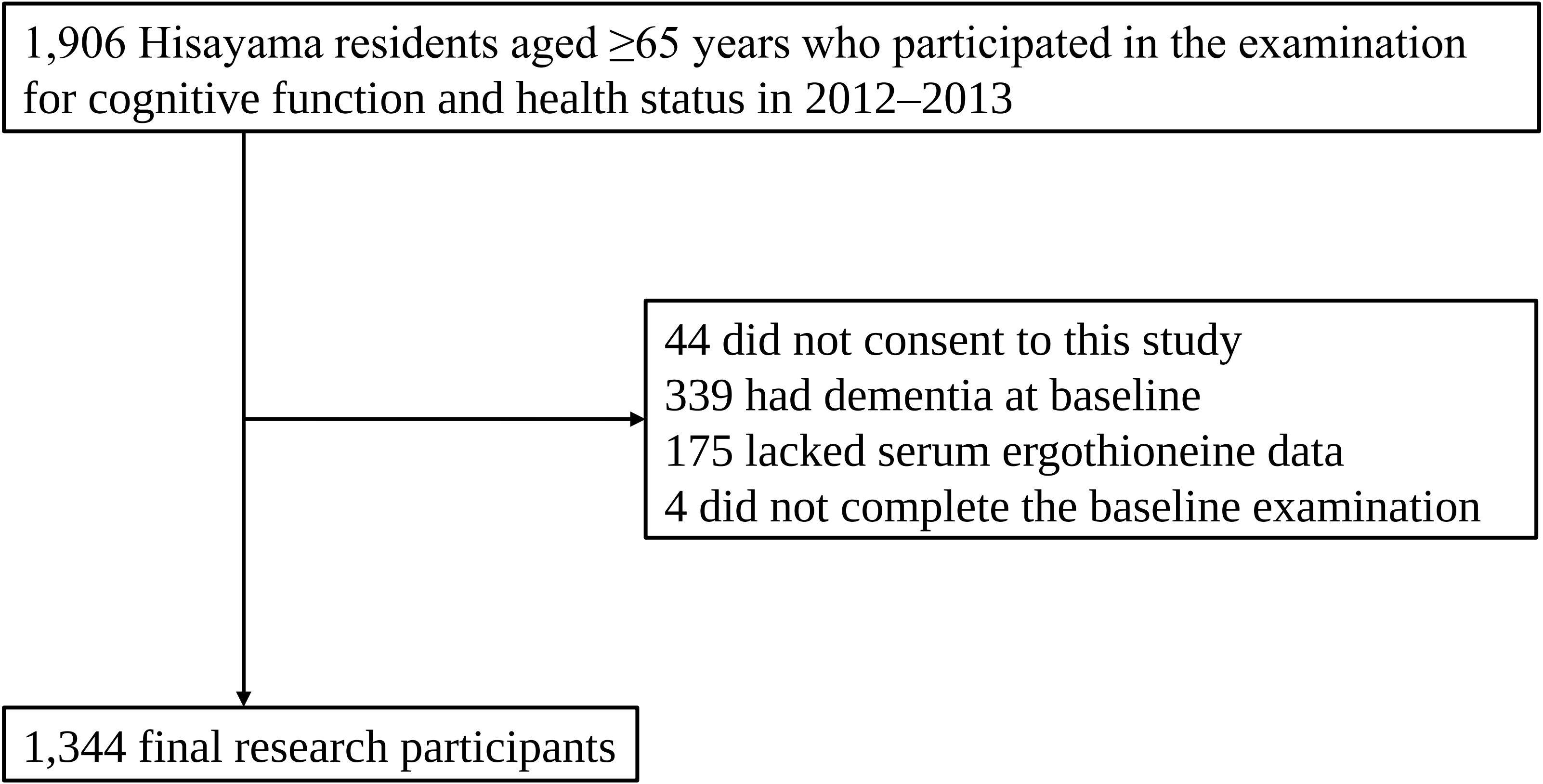
1,906 Hisayama residents aged ≥65 years who participated in the examination for cognitive function and health status in 2012–2013
44 did not consent to this study
339 had dementia at baseline
175 lacked serum ergothioneine data
4 did not complete the baseline examination
1,344 final research participants
