# Supplementary figures and images for: Serum ergothioneine and risk of dementia in a general older Japanese population: the Hisayama Study
Source: Psychiatry Clin Neurosci. 2025 Sep 5;79(12):808–16. doi: 10.1111/pcn.13893 (PMC12683611; doi:10.1111/pcn.13893)

Age- and sex-adjusted cumulative incidence  
of all-cause dementia, %

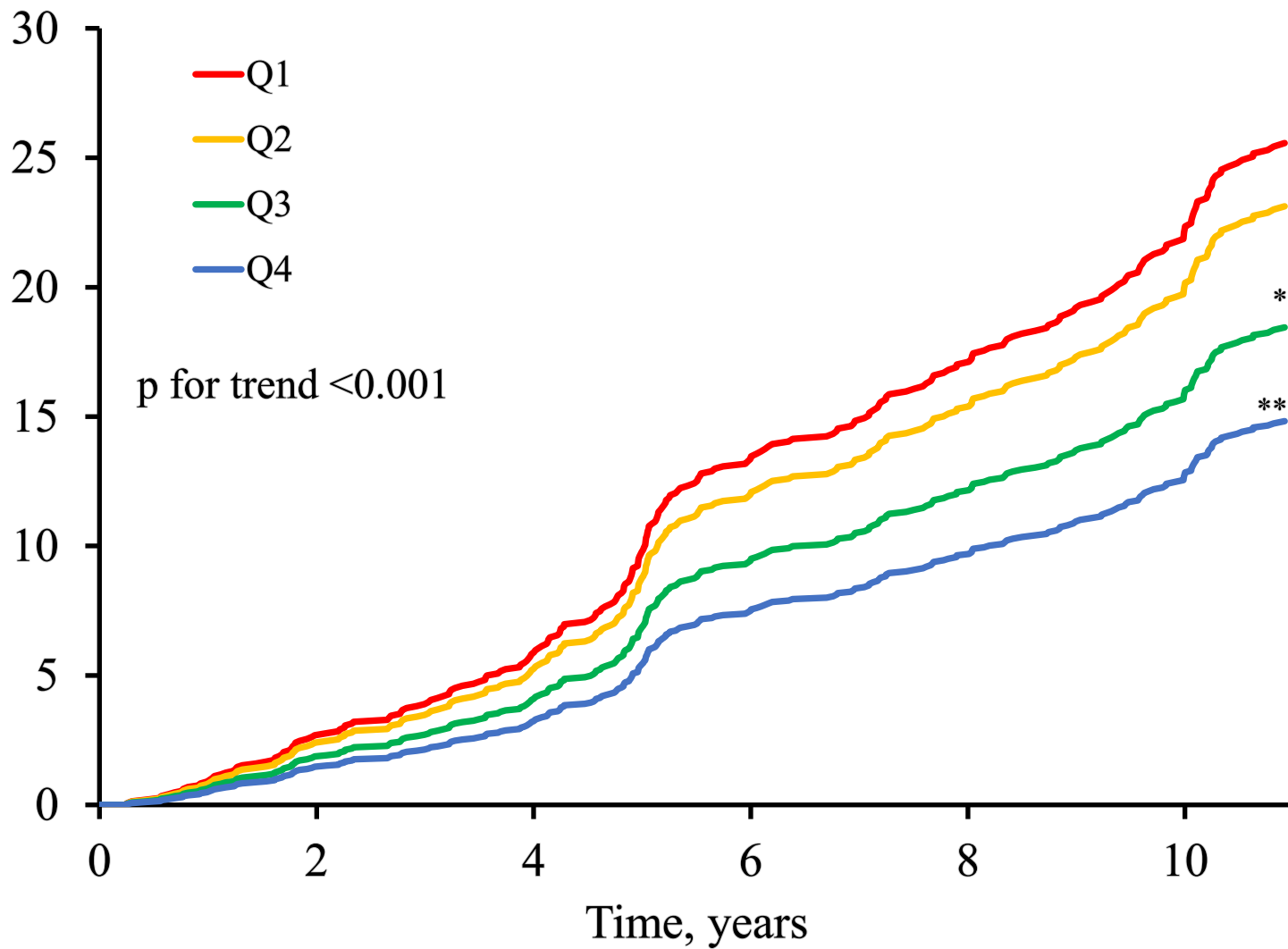

Supplement: Supplementary file 2 — Figure S2. Age‐ and sex‐adjusted cumulative incidence of all‐cause dementia according to quartiles of serum ergothioneine levels. [file PCN-79-808-s003.pdf]
