# Supplementary material for: Serum ergothioneine and risk of dementia in a general older Japanese population: the Hisayama Study
Source: Psychiatry Clin Neurosci. 2025 Sep 5;79(12):808–16. doi: 10.1111/pcn.13893 (PMC12683611; doi:10.1111/pcn.13893)
Supplement: Supplementary file 3 — Table S1. Sensitivity analyses of the association between serum ergothioneine levels and the risk of all‐cause dementia. [file PCN-79-808-s004.docx]

| Table S1. Sensitivity analyses of the association between serum ergothioneine levels and risk of dementia, 2012–2023 | | | | | | | | | | |
| --- | --- | --- | --- | --- | --- | --- | --- | --- | --- | --- |
| Serum ergothioneine levels (μmol/L) | Persons at risk, n | Events, n | Hazard ratio (95% confidence interval) | | | | | | | |
|  |  |  | Model 1  (Age- and sex-adjusted) | p for trend |  | Model 2  (Multivariable-adjusted^†^) | p for trend |  | Model 3  (Multivariable-adjusted^‡^) | p for trend |
| ***Sensitivity analysis when censoring participants who developed dementia during the initial 2 years of follow-up*** | | | | | | | | | | |
| Q1 (<0.410) | 335 | 76 | 1.00 (reference) |  |  | 1.00 (reference) |  |  | 1.00 (reference) |  |
| Q2 (0.410–0.692) | 337 | 66 | 0.91 (0.65–1.27) |  |  | 0.97 (0.69–1.35) |  |  | 0.95 (0.66–1.37) |  |
| Q3 (0.693–1.229) | 335 | 50 | 0.73 (0.51–1.05) |  |  | 0.76 (0.53–1.10) |  |  | 0.78 (0.53–1.14) |  |
| Q4 (>1.229) | 337 | 42 | 0.60 (0.41–0.88) | 0.004 |  | 0.61 (0.41–0.91) | 0.008 |  | 0.61 (0.40–0.93) | 0.01 |
| ***Sensitivity analysis accounting for competing risks of death using the Fine–Gray subdistribution hazards model*** | | | | | | | | | | |
| Q1 (<0.410) | 335 | 92 | 1.00 (reference) |  |  | 1.00 (reference) |  |  | 1.00 (reference) |  |
| Q2 (0.410–0.692) | 337 | 78 | 0.91 (0.67–1.24) |  |  | 0.93 (0.67–1.28) |  |  | 0.93 (0.66–1.32) |  |
| Q3 (0.693–1.229) | 335 | 57 | 0.71 (0.50–0.99) |  |  | 0.69 (0.49–0.97) |  |  | 0.72 (0.50–1.04) |  |
| Q4 (>1.229) | 337 | 46 | 0.54 (0.38–0.78) | <0.001 |  | 0.53 (0.37–0.77) | <0.001 |  | 0.55 (0.37–0.82) | 0.002 |
| ^†^ Model 2: Adjusted for age, sex, education status, systolic blood pressure, antihypertensive medication, diabetes mellitus, serum total cholesterol, body mass index,  electrocardiogram abnormalities, history of stroke, smoking habits, alcohol intake, and regular exercise.  ^‡^ Model 3: Adjusted for the covariates included in model 2 plus daily vegetable intake. | | | | | | | | | | |
