# Supplementary material for: Serum ergothioneine and risk of dementia in a general older Japanese population: the Hisayama Study
Source: Psychiatry Clin Neurosci. 2025 Sep 5;79(12):808–16. doi: 10.1111/pcn.13893 (PMC12683611; doi:10.1111/pcn.13893)
Supplement: Supplementary file 4 — Table S2. Association between serum ergothioneine levels and risk of all‐cause death. [file PCN-79-808-s001.docx]

| Table S2. Association between serum ergothioneine levels and risk of all-cause death, 2012–2023 | | | | | | | | | | |
| --- | --- | --- | --- | --- | --- | --- | --- | --- | --- | --- |
| Serum ergothioneine levels (μmol/L) | Persons at risk, n | Events, n | Hazard ratio (95% confidence interval) | | | | | | | |
|  |  |  | Model 1  (Age- and sex-adjusted) | p for trend |  | Model 2  (Multivariable-adjusted^†^) | p for trend |  | Model 3  (Multivariable-adjusted^‡^) | p for trend |
| Q1 (<0.410) | 335 | 129 | 1.00 (reference) |  |  | 1.00 (reference) |  |  | 1.00 (reference) |  |
| Q2 (0.410–0.692) | 337 | 100 | 0.76 (0.58–0.99) |  |  | 0.85 (0.65–1.11) |  |  | 0.85 (0.63–1.14) |  |
| Q3 (0.693–1.229) | 335 | 87 | 0.78 (0.60–1.03) |  |  | 0.87 (0.66–1.15) |  |  | 0.83 (0.61–1.13) |  |
| Q4 (>1.229) | 337 | 82 | 0.77 (0.58–1.01) | 0.06 |  | 0.80 (0.60–1.06) | 0.14 |  | 0.78 (0.57–1.07) | 0.13 |
| ^†^ Model 2: Adjusted for age, sex, education status, systolic blood pressure, antihypertensive medication, diabetes mellitus, serum total cholesterol, body mass index,  electrocardiogram abnormalities, history of stroke, smoking habits, alcohol intake, and regular exercise.  ^‡^ Model 3: Adjusted for the covariates included in model 2 plus daily vegetable intake. | | | | | | | | | | |
