# Supplementary material for: Serum ergothioneine and risk of dementia in a general older Japanese population: the Hisayama Study
Source: Psychiatry Clin Neurosci. 2025 Sep 5;79(12):808–16. doi: 10.1111/pcn.13893 (PMC12683611; doi:10.1111/pcn.13893)
Supplement: Supplementary file 5 — Table S3. Association between serum ergothioneine levels and risk of all‐cause dementia stratified by MCI status at baseline, 2012–2023. [file PCN-79-808-s002.docx]

| Table S3. Association between serum ergothioneine levels and risk of all-cause dementia stratified by MCI status at baseline, 2012–2023 | | | | | | | | | | |
| --- | --- | --- | --- | --- | --- | --- | --- | --- | --- | --- |
|  |  |  | Hazard ratio (95% confidence interval) | | | | | | | |
| Serum ergothioneine  levels (μmol/L) | No. of events/PYs | Crude  incidence rate (per 10^3^ PYs) | Model 1 (Age- and sex-adjusted) | p for  trend |  | Model 2 (Multivariable-adjusted^†^) | p for  trend |  | Model 3 (Multivariable-adjusted^‡^) | p for  trend |
| Participants without MCI |  |  |  |  |  |  |  |  |  |  |
| Q1 (<0.410) | 65/2645 | 24.6 | 1.00 (reference) |  |  | 1.00 (reference) |  |  | 1.00 (reference) |  |
| Q2 (0.410–0.692) | 59/2851 | 20.7 | 0.96 (0.67–1.37) |  |  | 1.00 (0.70–1.44) |  |  | 1.11 (0.75–1.65) |  |
| Q3 (0.693–1.229) | 47/3024 | 15.5 | 0.79 (0.54–1.15) |  |  | 0.80 (0.55–1.18) |  |  | 0.87 (0.58–1.30) |  |
| Q4 (>1.229) | 34/3161 | 10.8 | 0.56 (0.37–0.85) | 0.004 |  | 0.56 (0.36–0.86) | 0.005 |  | 0.62 (0.39–0.99) | 0.03 |
| Participants with MCI |  |  |  |  |  |  |  |  |  |  |
| Q1 (<0.410) | 27/362 | 74.6 | 1.00 (reference) |  |  | 1.00 (reference) |  |  | 1.00 (reference) |  |
| Q2 (0.410–0.692) | 19/366 | 51.9 | 0.74 (0.41–1.34) |  |  | 0.68 (0.36–1.30) |  |  | 0.55 (0.26–1.14) |  |
| Q3 (0.693–1.229) | 10/330 | 30.3 | 0.47 (0.23–0.98) |  |  | 0.37 (0.16–0.83) |  |  | 0.32 (0.12–0.81) |  |
| Q4 (>1.229) | 12/282 | 42.6 | 0.55 (0.28–1.10) | 0.04 |  | 0.60 (0.28–1.28) | 0.06 |  | 0.47 (0.20–1.09) | 0.04 |
| Abbreviations: MCI, mild cognitive impairment; PYs, person-years. ^†^ Model 2: Adjusted for age, sex, education status, systolic blood pressure, antihypertensive medication, diabetes mellitus, serum total cholesterol, body mass index, electrocardiogram abnormalities, history of stroke, smoking habits, alcohol intake, and regular exercise. ^‡^ Model 3: Adjusted for the covariates included in model 2 plus daily vegetable intake. | | | | | | | | | | |
